# Clinical and genetic peculiarities of vasculitis associated with Familial Mediterranean fever in Armenian children

**DOI:** 10.1186/1546-0096-13-S1-P91

**Published:** 2015-09-28

**Authors:** G Amaryan, T Sarkisian, A Tadevosyan

**Affiliations:** 1 “Arabkir” Medical Centre-Institute of Child and Adolescent Health; Yerevan State Medical University, National Paediatric Centre for Familial Mediterranean Fever, Yerevan, Armenia; 2 Yerevan State Medical University, Pediatrics, Yerevan, Armenia; 3 Yerevan State Medical University, Medical Genetics, Yerevan, Armenia; 4 Centre of Medical Genetics and Primary Health Care, Medical Genetics, Yerevan, Armenia; 5 Yerevan State Medical University, Public Health and Health Care, Yerevan, Armenia

## Introduction

Familial Mediterranean fever (FMF) is the most common hereditary disorder among Armenians. It manifests mainly in childhood and represents a significant health care pediatric problem. The clinical picture of FMF and vasculitis have much in common: fever, abdominal pain, arthritis, myalgia, skin lesions. Numerous data indicate a higher incidence of vasculitis in FMF patients, compared with healthy ethnically matched populations.

## Objective

To investigate clinical and genetic peculiarities of vasculitis associated with FMF in children in Armenia.

## Methods

A group of 715 children with FMF was observed at the National Pediatric Centre for FMF (438 boys, 277 girls, mean age 8.64±0.17). The diagnosis of FMF was confirmed based on the Tel-Hashomer criteria and molecular genetic detection of MEFV mutations. For statistical analysis standard statistical Epi-Info 2000 Program was performed.

## Results

Frequency of vasculitis in Armenian children with FMF was rather high - 4.3% (31 children). Henoch-Shonlein Purpura (HSP) was diagnosed in 1.5% (11) patients, Protracted Febrile Myalgia (PFM) - in 2.7% (20). FMF in these patients characterized by early onset (mean age 3 years), high (4 fold) risk of PFM [RR = 3.90 (1.32 ÷ 11.35); χ2 = 5.94; p = 0.015], as well as late diagnosis of FMF (9.42 ± 0.72) and late onset of colchicine treatment. They had also high frequency of severe FMF attacks, prevalence of acute recurrent arthritis and HSP and PFM manifestation after 5-6 year of FMF onset. The risk of HSP was 5-fold increased in children with severe FMF compared with moderate activity of disease. The development of vasculitis was associated with M694V-homozygous and compound-heterozygous genotypes. Particularly, HSP and PFM were observed respectively at 2.9% and 4.6% M694V-homozygous patients (χ2 = 8.27; p <0.02), which confirms the influence of *MEFV* genotype on the development of vasculitis.

## Conclusion

Armenian children with FMF had higher than expected frequency of vasculitis (4.3%). We suppose, that in children with FMF: 1) HSP and PFM vasculitis might be considered as markers of severe FMF and early disease onset 2) M694V homozygous genotype is a risk factor for the development of PFM. These results are consistent with data on the susceptibility of FMF patients in ethnically matched populations in the development of HSP and PFM *[Lange-Sperandio B. et al., 2004].*MEFV mutation genetic screening is recommended for Armenian children with HSP, PFM vasculitis for early diagnosis of FMF, treatment and prevention of complications.

